# Separation of Radio-Frequency Sources and Localization of Partial Discharges in Noisy Environments

**DOI:** 10.3390/s150509882

**Published:** 2015-04-27

**Authors:** Guillermo Robles, José Manuel Fresno, Juan Manuel Martínez-Tarifa

**Affiliations:** Department of Electrical Engineering, University Carlos III of Madrid, Avda. Universidad, 30, Leganés 28911, Spain; E-Mails: jfresno@ing.uc3m.es (J.M.F.); jmmtarif@ing.uc3m.es (J.M.M.-T.)

**Keywords:** partial discharges, spectral power, Condition Monitoring, RF Measurements, RF Localization, particle swarm optimization

## Abstract

The detection of partial discharges (PD) can help in early-warning detection systems to protect critical assets in power systems. The radio-frequency emission of these events can be measured with antennas even when the equipment is in service which reduces dramatically the maintenance costs and favours the implementation of condition-based monitoring systems. The drawback of these type of measurements is the difficulty of having a reference signal to study the events in a classical phase-resolved partial discharge pattern (PRPD). Therefore, in open-air substations and overhead lines where interferences from radio and TV broadcasting and mobile communications are important sources of noise and other pulsed interferences from rectifiers or inverters can be present, it is difficult to identify whether there is partial discharges activity or not. This paper proposes a robust method to separate the events captured with the antennas, identify which of them are partial discharges and localize the piece of equipment that is having problems. The separation is done with power ratio (PR) maps based on the spectral characteristics of the signal and the identification of the type of event is done localizing the source with an array of four antennas. Several classical methods to calculate the time differences of arrival (TDOA) of the emission to the antennas have been tested, and the localization is done using particle swarm optimization (PSO) to minimize a distance function.

## 1. Introduction

The correct maintenance of high voltage equipment is an advantageous strategy for electric utilities to avoid unexpected outages [1,2]. Among the techniques used to make a diagnosis of electric machines and power cables, the measurement of partial discharges (PD) is one of the most versatile. Partial discharges are low energy ionizations highly localized in certain electrical insulation volumes. They can be a consequence of different imperfections in the systems, like sharp energized metallic objects, highly contaminated insulators and gaseous voids within liquid/solid insulation systems [3–5]. Thus, PD measurements can reveal multiple ageing mechanisms in high voltage infrastructures [4–6]. Partial discharge sources can be identified through the well-known PD phase resolved (PRPD) patterns, which represent PD magnitudes referenced to the applied high voltage. This is achieved through voltage dividers and high frequency current transformers (HFCT) [5,7,8]. In a further step, in order to promote the application of this technique for on-line measurements, acoustic and radio frequency (RF) emissions from PD are being measured using ultrasonic sensors [9,10] and antennas [11,12], respectively. In addition, these techniques allow making PD location using arrays of sensors simultaneously, which is especially useful inside oil-insulated power transformers and gas-insulated substations and in air-insulated substations [13]. However, when high levels of noise or interferences are present, the lack of synchronization signal makes it difficult to identify the PD pulses and determine if there is PD activity. Some previous works have been focused on PD identification through their pulse spectra, but the results seem to be still restricted to laboratory environments [14–16], or very specific confined environments such as gas insulated substations (GIS) [17,18]. In this paper, the authors address the problem from a totally different point of view since the identification of the source is not totally based on the spectral characteristics of the emitted signal. The method combines a pulse separation technique to isolate different simultaneous events with a geometric location algorithm to identify the pulse sources. In particular, the spectral power ratios (PR) maps presented by the authors in previous papers [8,19], separate the pulse sources. Then, once the pulse sources are selected through their spectra, the time-differences-of-arrivals (TDOA) measured by four antennas are used to set an objective function that, when minimized, gives the coordinates of the pulse source. In order to obtain accurate results for the calculation of the TDOA, several methods based on cross correlation and cumulative energy of the signals have been tested. The objective function is minimized using the particle swarm optimization (PSO) algorithm giving excellent results in the geometric location.

## 2. Experimental Setup

**Figure 1:**
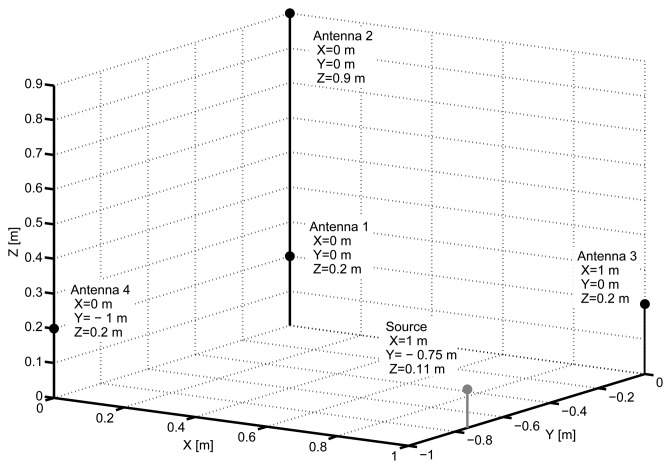
Antennas and first PD emitter position.

The main components of the setup are four antennas to localize the radio-frequency emission of the partial discharge pulses connected to a high-speed acquisition system, different partial discharge emitters connected to a high-voltage transformer and several disturbance sources. The antennas are simple monopoles 10 cm in length with good receptivity in the frequency range of the PD and interference emissions [20]. They were placed at four vertices of a tetrahedron with the base at 20 cm from the ground level, Figure 1, and connected to an oscilloscope through coaxial cables 5 m long. The oscilloscope bandwidth is 2.5 GHz and the sampling frequency was set to 5 GS/s so every sample was taken every 200 picoseconds. The time window is 1 microsecond so the signals have 5000 samples.

All partial discharges were generated with two objects connected independently. The first object is done by placing a non-insulated wire loop connected to ground around the insulation of a 25 kV high-voltage cable. When the cable is connected to a high-voltage source, a Schleich BV 702210 transformer with a GLP1-e HV control module, there is a high electric field gradient ring around the cable that triggers surface partial discharges for voltages above 3.5 kV. It is very common to find these type of PD on contaminated surfaces of insulators of transmission lines and bushings in substations though the true nature of the discharge is not relevant for the results obtained in this paper. The second object is a void created inside a methacrylate disk that generates internal partial discharges. The external electric field is applied placing the disk between two electrodes connected to high-voltage and ground, respectively. In order to avoid surface discharges, the electrodes and the disk are immersed in transformer oil.

**Figure 2:**
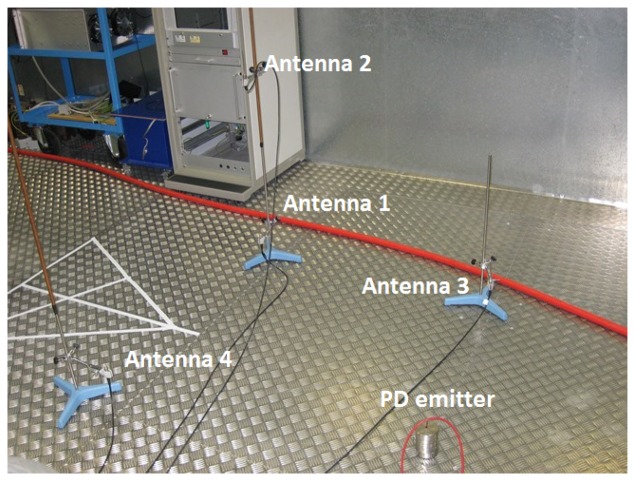
Setup in laboratory. Notice the abundance of metallic surfaces and structures around the antennas.

The activity of these discharges was previously confirmed in a phase-resolved partial discharge pattern (PRPD) drawn with a commercial PD acquisition system. The shape and geometry of the objects provide a direct path to the antennas mainly through air. Under this assumption, the speed of propagation will be *c* = 3 × 10^8^ ms^−1^, so the space resolution between samples is *d_min_* = 200 × 10^−12^ × 3 × 10^8^ = 0.06 m. The interferences are all radio and TV broadcasting emission, WiFi and mobile communications which, in most cases, are below the level of partial discharge pulses, and forced interferences coming from the arcing of fluorescent lamps that have peaks notably larger than the peaks of PD. Notice that there are many metallic structures around the setup, see Figure 2, so the multipath propagation of the signals due to reflections is very important and in many cases compromise the use of cross-correlation methods to calculate the time differences of arrival to the antennas.

## 3. Signal Processing

The data are postprocessed to separate sources and, therefore, to distinguish partial discharges from interferences. The identification could be easily done in a PRPD pattern with the information of the phase of a voltage reference, but this signal is usually missing in radio-frequency measurements. The alternative proposed in this paper is the separation of radio-frequency sources through the spectral characteristics of the signals. These characteristics are defined by the power ratio (PR) parameters which represent the power share of the pulses in two bands of frequency. Once the pulses have been separated in clusters it is possible to analyze them separately to calculate the location of the source and to determine whether the pulses are partial discharges or interferences.

### 3.1. Introduction to Power Ratios Mapping

Many signal processing techniques based on the spectral differences between pulses have been applied to HF/VHF waveforms detected with inductive devices to identify partial discharges when noise is present [8]. These differences have also been observed in the UHF range [14–18], so the spectral analysis of these emissions can help in the characterization of different sources of noise and PD. In this paper, the authors will use the analysis of the share of spectral power applied to RF signals, since it has already given good separation results between noise and PD sources in the HF/VHF range [8,19].

The presented technique calculates the accumulated spectral power of all signals in two frequency intervals; these values are normalized to the total spectral power of the signal, to obtain relative magnitudes of power non-dependent on the signal amplitudes. Thus, each pulse will have two power ratios (PR), whose values can be represented in a two-dimensional map to help the system user in the characterization of each source. These parameters are characterized by the frequency intervals where the spectral power is calculated, so they will be referred as power ratio for low frequencies (PRL) and power ratio for high frequencies (PRH), and they are defined as follows:

(1) $$\text{PRL}=\frac{\sum_{f_{1L}}^{f_{2L}}{\left|s\left(f\right)\right|}^{2}}{\sum_{0}^{f_{t}}{\left|s\left(f\right)\right|}^{2}}\times 100$$

(2) $$\text{PRH}=\frac{\sum_{f_{1H}}^{f_{2H}}{\left|s\left(f\right)\right|}^{2}}{\sum_{0}^{f_{t}}{\left|s\left(f\right)\right|}^{2}}\times 100$$

Being |*s*(*f*)|, the magnitude of the fast Fourier transform of the pulse *s*(*t*). The frequencies *f*_1_*_L_*, *f*_2_*_L_*, *f*_1_*_H_*, *f*_2_*_H_*, and *f_t_* are configurable by the user according to the characteristics of *s*(*f*), complying with the conditions 0 ≤ *f*_1_*_L_* < *f*_2_*_L_*, *f*_1_*_H_* < *f*_2_*_H_* ≤ *f_t_* and *f*_1_*_L_* < *f*_2_*_H_*. The results from previous works reveal that each pulse source (whether it is noise or PD) will show a characteristic cluster, which usually can be distinguished from the rest.

The position and shape of the clusters will also depend on the selected intervals, which can be separated or overlapped. If the calculation intervals are narrow, the power ratio will be small and the clusters will be confined close to the axes of the map. If the intervals are wide and complementary (half of the total bandwidth for each one, for example), the represented information will be redundant because *PRH* = 100 − *PRL*. Thus, for the same test object, some frequency intervals may give a better separation of the clusters than others. In most of the cases, a simple selection using one third of *f_t_* for each interval results in an appropriate source separation [19]. Otherwise, the intervals should be chosen based on the observation of the on-line acquisition of the pulses spectrum [21].

### 3.2. Time Differences of Arrival

Once the pulses have been separated, the data from the clusters is analyzed to determine the location of the source. This is done firstly, calculating the time-differences-of-arrival (TDOA) of the signal to the four antennas and, then, minimizing an objective function. The first step is critical in the process [22], because small errors in the TDOA can give locations several centimeters or even meters apart from the real location. The algorithm that calculates the TDOA has to be unsupervised so no human interaction is needed. The main causes of error are due to unclear wavefronts that can be hidden in noise or contaminated with interferences with high-frequency components. In addition, obstacles in the direct path to the antennas can attenuate and hinder the wavefront and reflections in the surrounding metallic structures can create positive interferences that give peaks long past the first front. Therefore, a special effort was put in this stage of the algorithm to obtain the most accurate solutions and several methods to obtain TDOA were tested. In this study, the position of the source is known so we can evaluate the results of the different algorithms when compared with the real solution.

#### 3.2.1. Cross Correlation

The classical method to determine the time shift between two signals, *s*_1_ and *s*_2_, is calculating their cross correlation, *R*_12_(*n*), and finding the offset of the maximum of this function.

(3) $$R_{12}\left(n\right)=\sum_{m=-N/2}^{N/2-1}s_{1}\left(m+n\right)s_{2}\left(m\right)$$

where *N* is the total number of samples. However, the nature of radio frequency signals, where the reflections due to multipath propagation can have larger peaks than the direct frontwave, hinders the calculation of the TDOA. Even using preprocessors of the generalized correlation method [23], such as the Roth processor, the smoothed coherence transform (SCOT) and the phase transform (PHAT) did not help in obtaining good results.

**Figure 3:**
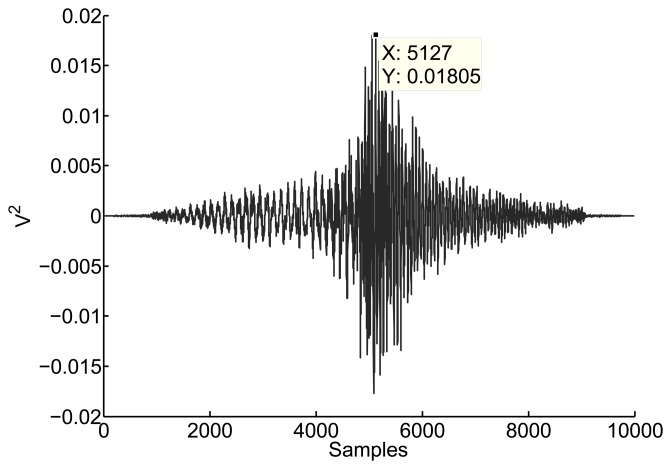
Cross correlation between two RF signals. The maximum is located at 5127 samples, so the time separation between signals is 127 samples, 25.4 nanoseconds or 7.62 m.

Figure 3 shows the cross correlation between two signals received in two antennas separated 61.2 cm. The error introduced by this method is quite notable since the distance in samples should be 10.2 instead of 127.

#### 3.2.2. Cumulative Energy

The cumulative energy of the signal up to sample *n*, [24], is calculated with:

(4) $$E_{c}\left(n\right)=\sum_{m=0}^{n}s^{2}\left(m\right)$$

**Figure 4:**
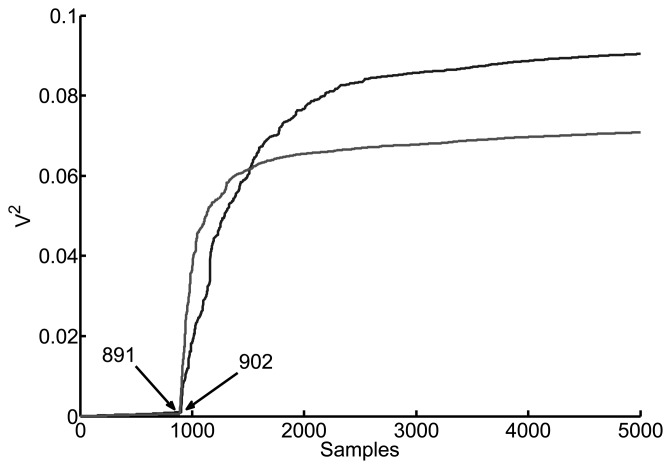
Cumulative energy of the same signals of Figure 3. Notice that the actual shift between them is only 11 samples instead of 127.

A typical plot of the energy of the same signals used before is shown in Figure 4. The accuracy of this method for the same two test signals is noticeably better obtaining an error of less than 1 sample, 11 instead of 10.2. In general, first, there is a continuous accumulation with a small slope due to noise and small interferences and then a dramatic change in slope when the signal begins. Unfortunately, this change of slope is sometimes difficult to define even using the maximum derivative of the cumulative energy curve. In the case of multipath reflections the slope can be steeper after the correct initial time and the algorithm should check whether the pulse has already started or not. For this reason, finding the exact instant of time where the signals start can be arduous and in most occasions there is an uncertainty of several samples that will turn out to be critical.

#### 3.2.3. Cumulative Energy with Negative Slope

**Figure 5:**
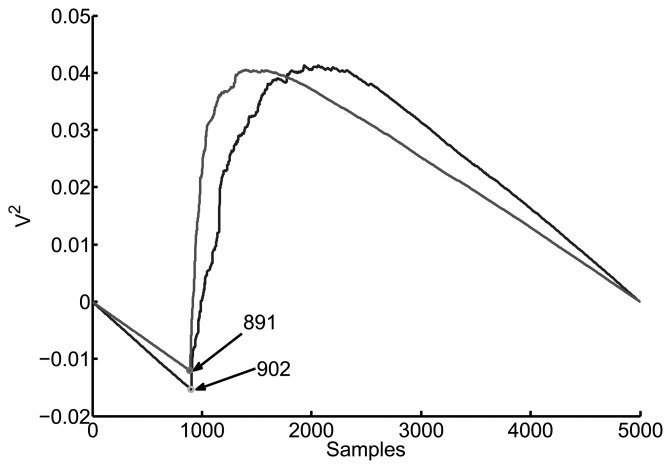
Minimum cumulated energy of the same signals as in Figures 3 and 4. This method also obtains a shift of 11 samples between them.

Modifying Equation (4) adding a line with negative slope can greatly simplify the calculation of the TDOA:

(5) $$E_{\text{neg}}\left(n\right)=E_{c}\left(n\right)-n\frac{E_{N}}{N}=\sum_{m=0}^{n}\left(s^{2}\left(m\right)-n\frac{E_{N}}{N}\right)$$

where *E_N_* is the total energy of the signal. Figure 5 shows the cumulative energy with negative slope for the same signals as before. The absolute minimum is taken as the start of the pulse so there is no need to find where the curve bends. So far, this is the method that has given the most accurate results for all types of signals.

#### 3.2.4. Akaike Information Criterion (AIC)

This method considers the signals as autoregressive processes in which every sample is a linear combination of past values. A quantity called Akaike information criterion (AIC) measures how well a statistical model fits a set of observations. This idea can be applied to determine the onset time of a signal by comparing two of its segments from sample 1 to *n* and from *n* + 1 to *N* [25]. The AIC is defined as,

(6) $$\text{AIC}\left(n\right)=n\times \ln\left[\sigma^{2}\left(1,n\right)\right]+\left(N-n-1\right)\times \ln\left[\sigma^{2}\left(n+1,N\right)\right]$$

where

$$\sigma^{2}\left(1,n\right)=\frac{1}{n}\sum_{m=0}^{n}{\left[s\left(m\right)-\bar{s}\left(1,n\right)\right]}^{2}$$

(7) $$\sigma^{2}\left(n+1,N\right)=\frac{1}{N-n-1}\sum_{m=n+1}^{n}{\left[s\left(m\right)-\bar{s}\left(n+1,N\right)\right]}^{2}$$

are the variances of the samples in the segments and *s̄*(·) is the mean value of the signal in the considered interval.

**Figure 6:**
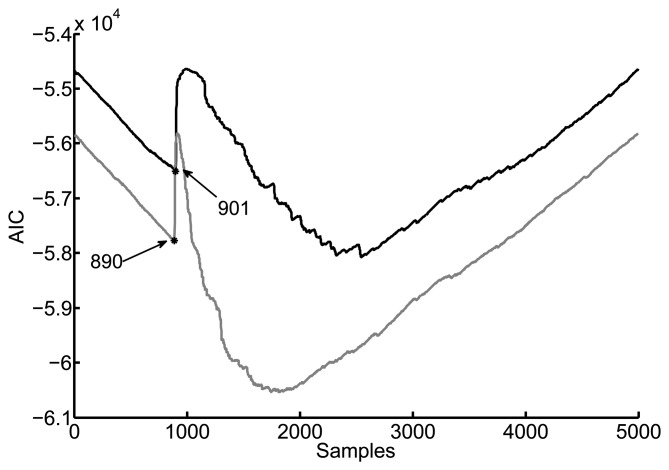
Onset determination with the AIC method using the same signals.

Figure 6 shows the performance of the Akaike information criterion for the same pair of signals. The TDOA is again 11 samples though the picking is found one sample earlier. Notice that the time of arrival has to be located in a window that encompasses closely the onset of the signal, otherwise, the minimum value of the AIC would be far from the correct solution. For this reason, the method applied in this paper has been the cumulative energy with negative slope.

### 3.3. Solving the Non-Linear Equation

Let the position of the source be **P_s_** = (*x_s_*,*y_s_*,*z_s_*), then, the distance to antenna *i* at **P_i_** = (*x_i_*,*y_i_*,*z_i_*) is $\left\|P_{i}-P_{s}\right\|=\sqrt{{\left(x_{i}-x_{s}\right)}^{2}+{\left(y_{i}-y_{s}\right)}^{2}+{\left(z_{i}-z_{s}\right)}^{2}}$. Therefore, the difference of distances between the source and the antennas *i* and *j* would be *D_ij_* = ‖**P_i_** − **P_s_**‖ − ‖**P_j_** − **P_s_**‖ or *D_ij_* — ‖**P_i_** — **P_s_**‖ + ‖**P_j_** — **P_s_**‖ = 0. In this equation, the unknowns are the coordinates of the source, so let **P̂_s_** = (*x̂_s_*, *ŷ_s_*, *ẑ_s_*) be the estimation of its position. Repeating the process for all pairs of antennas and summing all equations together gives:

(8) $$f\left({\hat{x}}_{s},{\hat{y}}_{s},{\hat{z}}_{s}\right)=\sum_{i-1}^{L-1}\sum_{j=i+1}^{L}{\left(D_{ij}-\left\|P_{\text{i}}-{\hat{P}}_{s}\right\|+\left\|P_{j}-{\hat{P}}_{s}\right\|\right)}^{2}$$

where *L* is the number of antennas and the distances difference have been squared to consider only positive values in the objective function. Equation (8) would be 0 for the correct estimation of **P̂_s_**, so any method that minimizes *f*(*x̂_s_*, *ŷ_s_*, *ẑ_s_*) would give the solution to the position of the source. This equation is similar to the function used in [26] though we consider only six TDOA.

#### Particle Swarm Optimization (PSO)

This method is a very simple algorithm that optimizes an objective function by placing a flock or swarm of entities in the solution space [27]. The initial deployment is random or, as in our case, a hexahedron in cartesian coordinates. In every iteration *l*, every particle evaluates the function in Equation (8) at its own position. The best solution of each individual *k*, **P_k,best_**(*l*) is registered and updated, as well as the best global solution of all the swarm, **P**_**best**_(*l*). These two parameters are part of the swarm intelligence so every particle knows its own best fitness and the particle with the best fitness. Their positions are changed according to this information so there will be a component in the velocity of particle *k* that ties the particle to its current best position and a component that moves the particle towards the global best [28]:

(9) $$\begin{array}{ll} v_{k}\left(l+1\right) & =v_{k}\left(l\right)+U_{1}\left(0,1\right)⊗\left[P_{k,\text{best}}\left(l\right)-P_{k}\left(l\right)\right]\\ & +U_{2}\left(0,1\right)⊗\left[P_{\text{best}}\left(l\right)-P_{k}\left(l\right)\right],\\ P_{k}\left(l+1\right) & P_{k}\left(l\right)+v_{k}\left(l+1\right). \end{array}$$

where **U_1_**(0, 1) and **U_2_**(0, 1) are line matrices with three elements uniformly distributed between 0 and 1 that limit the movement of the particles towards their own best and the swarm best, respectively. The operator ⊗ multiplies the random numbers by the cartesian coordinates component by component. In some occasions, Equations (9) are complemented with an inertia that multiplies the velocity and two correction factors that multiply the uniform distributions. In our case, these parameters are set to 1 because the results were accurate.

When the function to minimize in Equation (8) is below a threshold or when a predefined number of iterations is reached, the algorithm exits the loop and **P̂_s_** is set to **P_best_**.

## 4. Measurements

**Figure 7:**
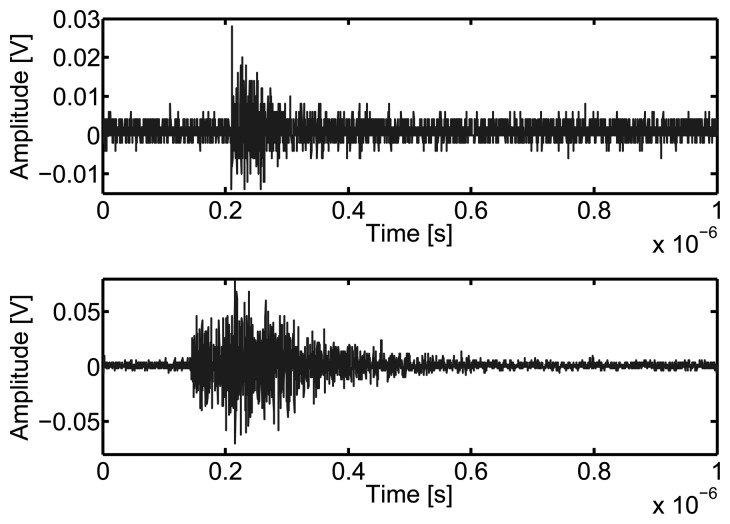
Partial discharge pulse (upper) and interference pulse (lower) acquired with monopole antennas 10 cm long.

All data are acquired after partial discharges have reached a stable activity at 11 kV for the first object and 14.8 kV for the second object. Then, all four channels of the oscilloscope are set to acquire 4 × 500 pulses that surpass the trigger threshold. These pulses can be either partial discharges or forced interferences because both sources are active simultaneously, see Figure 7.

The first step is to discriminate pulses created by partial discharges from pulsed interferences, in this case simulated with the arcing of fluorescent lamps. Even though PD are stochastic processes their frequency spectra have common characteristics that are directly related to the type of PD and the site where they are happening. This means that different sources will have different spectral components and finding the differences can be used to distinguish them. This is done by visual inspection of the Fourier transform of the incoming signals.

### 4.1. Surface Partial Discharges on a Wire

**Figure 8:**
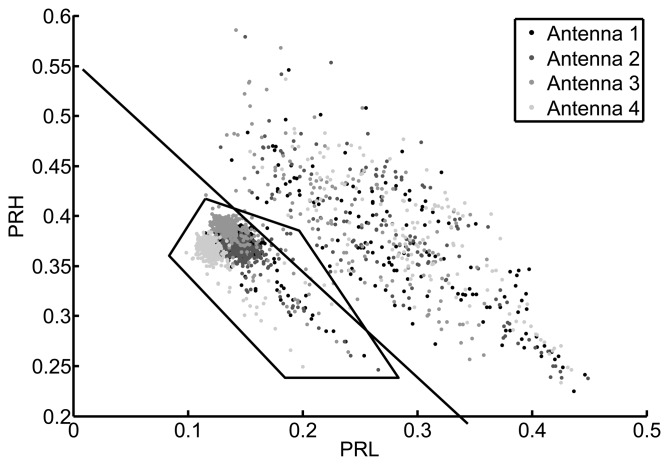
2D mapping of all pulses from the second object considering the low frequency interval in [50, 300] MHz and the high frequency interval in [600, 1600] MHz.

Figure 8 shows the PRL-PRH map for all measurements when the low frequency interval goes from 50 MHz to 300 MHz and the high frequency intervals spans from 600 Hz to 1600 MHz. At a first glance, the concentrated points could belong to a type of event while the scattered measurements would belong to another. However, the plot was divided by a line into two clusters attending exclusively at their separation in the map. The cluster above the line is more dispersive while the cluster below has a tight cloud and some dispersive points. Instead of selecting only the packed cluster, the selection area encompasses all points below the line and even some points that fell above the border. This careless choice of points is done to demonstrate the flexibility of the method when selecting the type of signals so, if some signals do not belong to the selected type, they will eventually be discarded in the process when they are found to be far from the most common solution.

**Figure 9:**
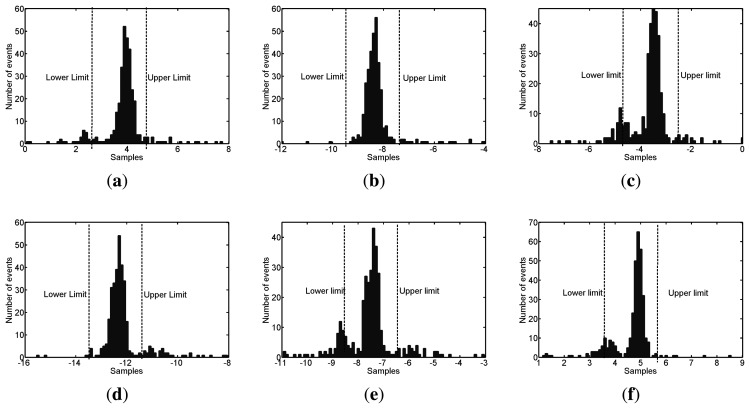
TDOA frequency distribution histograms for all pairs of antennas in the case of surface discharges. (**a**) Antennas 1 and 2; (**b**) Antennas 1 and 3; (**c**) Antennas 1 and 4; (**d**) Antennas 2 and 3; (**e**) Antennas 2 and 4; (**f**) Antennas 3 and 4.

The next step to find what type of pulse has been selected is to calculate the six TDOA of all four antennas using the method explained in Section 3.2.3. The signals are artificially oversampled by interpolating points using cubic splines between two real samples so the sampling time is reduced tenfold to 20 picoseconds. This decision helps in the accuracy of the TDOA calculations giving errors that are below the actual sampling time. However, even in the case that all selected points correspond to the same type of event there can be errors in the calculation of the TDOA. Therefore, the frequency distributions of the TDOA are used to select only the points that give a solution in the interval of the mode or plus-minus ten samples after oversampling, see Figure 9. In these plots, the abscissa axis represents the time-difference between two antennas in tens of samples considering that $T_{s}^{\prime }=20\;\text{ps}$. This is done to stress that there is a constraint in the time resolution limited by the sampling frequency. The ordinate axis is the number of events that give a certain TDOA. Assuming an error of ±10 samples is correct though it would induce an uncertainty in the possible geometric solutions.

For the sake of clearness, the histograms only include those points that give TDOA relatively close to the mode, they do not show all the points selected in Figure 8. Nevertheless, the points far from the mode would never be considered to calculate the position of the source. Table 1 contains the values of TDOA in samples calculated during the experiments. The first row are the actual time differences between the antennas and the position of the source. The second row are the average values of the time differences obtained after processing all signals and the third row are the most repetitive value of TDOA, the mode, for every pair of antennas. All values are very close together so the localization should be quite exact, though there are some errors. The last two rows are the absolute errors between the actual values and the average and mode and all of them are well below one sample.

**Figure 10:**
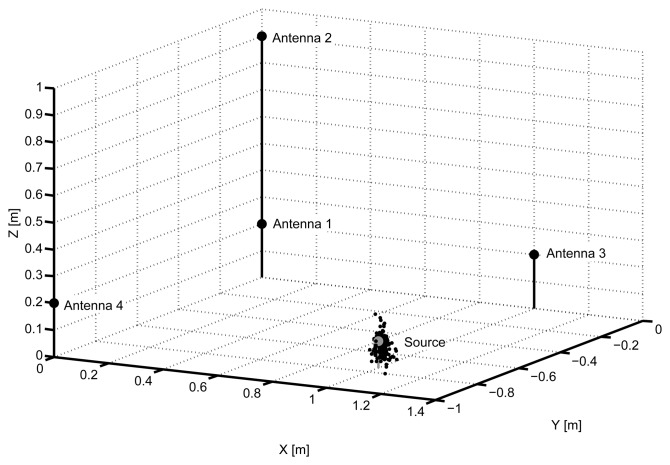
Solutions given by the particle swarm optimization algorithm for the first object.

The final step is to use the PSO algorithm to calculate the solutions for all the events selected in the histograms. Notice that a signal will be selected only if all the associated TDOA are inside the interval defined by the mode ±10 samples. If any of the TDOA falls outside that interval we consider that its calculation is wrong and the event is discarded. After running the PSO, the possible source positions are given in Figure 10 where it can be seen that they are very close to the actual partial discharges source. Additionally, Table 2 gives three different distances calculated from the actual position of the source to the average position of all solutions, **P̄_k_**, the mode of all solutions, namely, the most repetitive values of the components *x*, *y* and *z* of **P_k_**, and, finally, running the PSO algorithm only once using the mode of the six TDOA. In all cases, the error is close to 2 cm while the maximum resolution is 200 ps or 6 cm, which gives an idea of how accurate the results are.

In an open-air substation this would mean that all pulses falling below the line in Figure 8 are events that are occurring in a very specific spatial region. Localizing what is placed exactly on that position, by visual inspection or on the facility plan, will determine if they are partial discharges generated in that piece of equipment. Moreover, all points above the line can be discarded in future acquisitions to focus the attention in the really dangerous events. Once the partial discharges have been identified and the damaged asset localized, only one of the antennas would be necessary to continue with the supervision so an effective condition-based monitoring can be successfully conducted.

The number of particles searching for the solution has been set to 9^3^ = 729 though it has been found that similar solutions are obtained with less seeds, *i.e.*, 7^3^ = 343. The value of the objective function is calculated in every iteration and it has been found that 20 iterations are sufficient to reach an accurate solution. Every solution takes an average time of 0.25 seconds and the total runtime of the algorithm is 57 s.

### 4.2. Internal Discharges Inside a Dielectric

**Figure 11:**
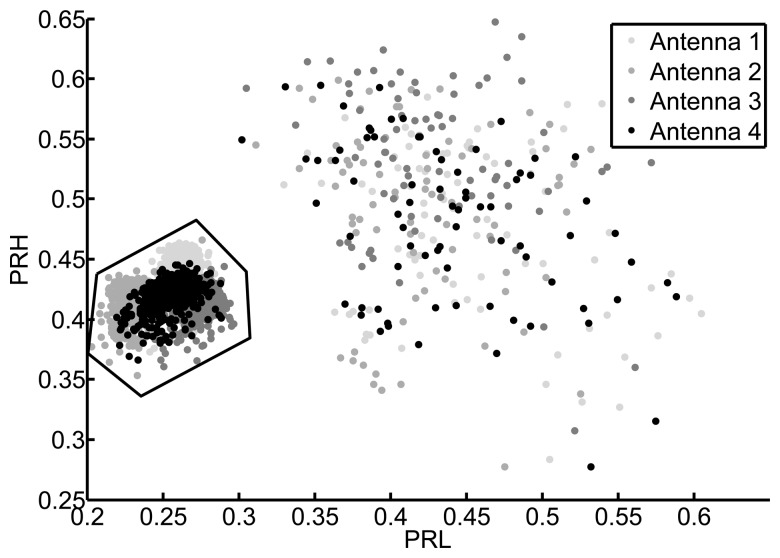
2D mapping of all pulses from the second object considering the low frequency interval in [50, 600] MHz and the high frequency interval in [400, 1300] MHz.

**Figure 12:**
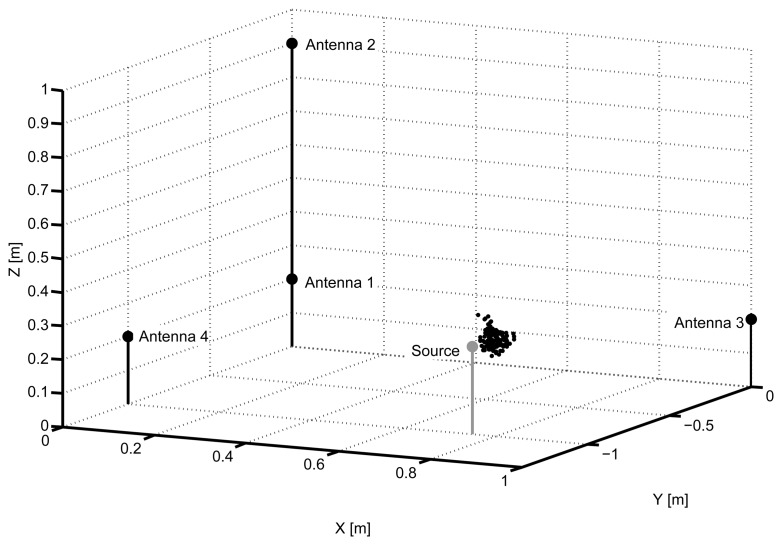
Solutions given by the particle swarm optimization algorithm for the second object.

All the processes are repeated using this second object in a different location. Figure 11 shows that the separation of events using the power ratio algorithm is more effective with this type of signals. The frequency intervals are from 50 MHz to 600 MHz in the case of the low frequency band (PRL) and from 400 MHz to 1300 MHz in the high frequency band (PRH). The packed cluster is selected to find its location and, after running the PSO algorithm, the distribution of possible solutions is shown in Figure 12. Notice that, though there is a small offset from the actual position of the source, its location can be clearly defined. Tables 3 and 4 give the values of the TDOA compared with the real one and the errors in distances, respectively.

## 5. Conclusions

Noise and interferences can make partial discharges undetectable. This problem is intensified when PRPD patterns are not available as happens in radio frequency measurements. Most methods devoted to the identification of PD are based on the study of the spectra of the pulses, but these methods will fail if the partial discharges are captured in noisy environments or if the pulses are distorted due to reflections in metallic parts. The method proposed in this paper separates all types of events captured with four antennas in clusters in two dimensional maps. Then, the signals in the clusters are analyzed by computing the time differences of arrival to all pairs of antennas. These TDOA can be used to locate geometrically the source in open air substations and, if the result is a specific point corresponding to an electrical asset, it would mean that there is a source of PD at that position. The tests conducted in the paper, show that partial discharges and noise interferences can be effectively separated with PR maps and the source of PD is accurately located. Then, once the cluster has been identified as derived from PD events, the system can filter out all other interferences and scan exclusively the selected signals.

## Figures and Tables

**Table 1:** Actual position of the source and solutions given by the PSO for the estimated TDOA.

	***t*_12_**	***t*_13_**	***t*_14_**	***t*_23_**	***t*_24_**	***t*_34_**
Real	3.758	−8.2976	−3.6423	−12.0556	−7.4003	4.6553
Average	3.6008	−8.2540	−3.6429	−11.8549	−7.2437	4.6111
Mode	3.900	−8.3000	−3.5000	−12.300	−7.4000	4.9000
Real – Average	0.1572	−0.0435	0.0006	−0.2007	−0.1566	0.0441
Real – Mode	−0.1420	0.0024	−0.1423	0.2444	−0.0003	−0.2447

**Table 2:** Distances from the calculated values to the actual position of the source.

	**Error (cm)**
**P̄_k_**	2.06
Mode of **P_k_**	2.16
**P_mode_**	2.03

**Table 3:** Actual position of the second object and solutions given by the PSO for the estimated TDOA.

	***t*_12_**	***t*_13_**	***t*_14_**	***t*_23_**	***t*_24_**	***t*_34_**
Real	2.5479	−3.6486	−8.3174	−6.1966	−10.8653	−4.6688
Average	1.9668	−4.3145	−7.5666	−6.2814	−9.5334	−3.2521
Mode	2.100	−4.200	−8.100	−6.300	−10.2000	−3.9000
Real – Average	0.5811	0.6659	−0.7508	0.0848	−1.3319	1.4167
Real – Mode	0.4479	0.5514	−0.2174	0.1034	−0.6653	−0.7688

**Table 4:** Distances from the calculated values to the actual position of the second object.

	**Error (cm)**
**P̄_k_**	6.25
Mode of **P_k_**	4.46
**P_mode_**	5.99
